# Changes of Mood and Cognitive Performance before and after a 100 km Nighttime Ultramarathon Run

**DOI:** 10.3390/ijerph17228400

**Published:** 2020-11-13

**Authors:** Daniel Krokosz, Ilona Bidzan-Bluma, Wojciech Ratkowski, Keqiang Li, Mariusz Lipowski

**Affiliations:** 1 Department of Psychology, Gdańsk University of Physical Education and Sport, 80-336 Gdansk, Poland; mariusz.lipowski@awf.gda.pl; 2 Institute of Psychology, University of Gdańsk, 80-309 Gdansk, Poland; bidzan.ilona@gmail.com; 3 Departament of Athletics and Motor Preparation, Gdańsk University of Physical Education and Sport, 80-336 Gdansk, Poland; wojciech.ratkowski@awf.gda.pl; 4 Faculty of Physical Culture, Gdańsk University of Physical Education and Sport, 80-336 Gdansk, Poland; keqiang.li@awf.gda.pl

**Keywords:** extreme-endurance sports, sport psychology, executive functions, psychology of running, cognition in sport

## Abstract

Ultramarathons are becoming an increasingly popular endurance sport. Year after year, the demands on athletes’ skills and endurance increase. Ultramarathons are particularly taxing on athletes’ psychological functioning. This study assessed the relationships between taking part in a nighttime ultramarathon and changes in mood and cognitive functioning. The study included 20 experienced runners aged 26–57 (M = 37.29; SD = 7.94) who had M = 7.08, SD = 5.41 (range 3–44) years of experience running. There were 18 men and 2 women. The mood states were measured twice, just before the start of the run and shortly after crossing the finish line, using the Polish version of the UMACL UWIST Mood Adjective Checklist by Mathews, Chamberlain, and Jones. To assess cognitive functioning, the Stroop Color and Word Test and “Forward digit span” subtest from the Wechsler Adult Intelligence Scale were used. We observed statistically significant changes in the mood of the runners: tense arousal, associated with the experienced stress, was significantly higher before the run than immediately after the finish. Moreover, we observed an improvement in cognitive functioning after finishing the 100 km run on both of the trials on the Stroop color word test and on the forward digit span test.

## 1. Introduction

Kaszubska Poniewierka (meanining in polish Cashubian ill-treatment) is a 100 km long cross-country ultramarathon. It takes place at night in the Pomeranian Voivodeship in Poland, mostly in forests and hills. The popularity of this race increases every year, alongside the numbers of people doing ultramarathons in Poland, Europe, and the United States. An ultramarathon is a run of any distance longer than 42.195 km (26.2 miles). However, the distances within this category vary greatly and depend on the race and the vision of its organizers, who often compete to organize the most extreme events. For example, races can take place in deserts at extremely high temperatures (e.g., Death Valley), in the Arctic, on high mountains, or simply with incredibly long distances. The longest ultramarathon to date had a distance of 4989 km and lasted 52 days—this means that participants had to run a distance greater than two marathons each day. It is worth noting that the average time for a professional runner to complete a marathon is around 2 h and 40 min in women and about 2 h and 10 min in men, while in the case of ultramarathon runs this time is about three times longer (approx. 8 h in women and 6 h 50 min in men) [1]. Studies show that marathoners differ from ultra-marathon runners by anthropometric characteristics [2], training speed (ultramarathon participants run slower) and experience with marathon running (UM runners have more finished marathon runs than marathon runners) [2,3] Furthermore, ultramarathon runners have greater pain tolerance than marathon runners [4]. As the popularity of these sports increases, the interest of researchers in this type of extreme endurance sport also increases. More and more people want to overcome the boundaries of their physical and mental endurance. It has been repeatedly shown that ultramarathons are associated with a significantly higher risk of dehydration, overexertion, or dysfunction of the gastrointestinal or urinary systems [5,6,7,8]. Obviously, these difficulties affect the mental wellbeing of runners, who are often focused on the internal struggle with these difficulties. Research on the psychological aspects of running ultramarathons is often prone to methodological difficulties stemming from the low number of individuals who agree to take part in such research, as doing so could make the already difficult task of running an ultramarathon even harder. As mentioned by Roebuck [9] in their meta-analysis of psychological research on ultramarathons, studies usually focus on personality features, motivation, mood, cognitive processes and functions, perception of pain, phenomena associated with psychological disorders such as addiction to physical exercise, the phenomenological aspects of running, or psychological interventions. This study focuses on the cognitive functioning of nighttime ultramarathon runners.

### 1.1. Mood in Ultramarathons

Results of the research on the mood of ultramarathon runners are consistent [9]. The overwhelming majority of studies found, unsurprisingly, that after running an ultramarathon, levels of vigor drop, and levels of mental and physical fatigue increase [10,11,12,13,14]. Our research group observed similar results in our previous study conducted during a 100 km run on a track. The UWIST Mood Adjective Check List revealed a clear drop in energy arousal 12 h after finishing the run [10]. Similarly, previous research suggests a post-race decrease in tension associated with pre-race stress [10,11,12,15]. Tension and pre-race stress are understandable in both beginner ultramarathoners, who have not previously finished races over 42.195 km, as well as in experienced runners who know what difficulties they will face and how unpleasant the consequences of finishing an ultramarathon can be. It is important to discuss to what extent mood during the run influences the runners’ results [16]. Undoubtedly, the emotions experienced by a runner can make it more difficult to concentrate on things important to a runner—especially those associated with positive self-talk, goal setting, and controlling one’s thoughts. However, it is also important to emphasize that sports psychology has recently come to emphasize that control over thoughts and emotions does not always lead to better performance, and it can itself be a factor leading to loss of focus on the task [13]. It is thus important to examine cognitive functions, including the short-term memory and executive functions of runners and their relationship with the runners’ moods.

### 1.2. Cognitive Functioning and Endurance Sports

Physical activity may influence cognitive functions as a result of the increase in the activity of the prefrontal cortex, which could translate into better results in terms of attention, memory, and executive functions [17]. Every sports discipline relies on cognitive and motor skills to different extents [18]. For example, in team sport such as volleyball, it seems that reaction time, executive control and perceptual speed are important [19], and cognitive functioning is dependent on the different competitive levels of players [20]. In the case of endurance sports, it seems that motor inhibition is one of the more important resources [18]. On the other hand, in the case of older individuals, endurance sports, including taking part in marathons, may foster the retention of cognitive functioning for a longer time. This especially concerns executive functions [21]—physical activity may be associated with less loss of brain tissue in areas associated with control skills [22]. However, there are some results showing that there is an improvement in the cognitive performance of elderly runners. [23]. However, in young and healthy individuals, the relationship between exercise and improvement in cognitive functions seems to be less pronounced [24]. Moreover, athletes appear to score better on scales which measure processing speed [25]. Arousal associated with doing sports may also improve or decrease performance during and/or after a workout. In the case of running, memory seems to worsen during running, but slightly improve after the run. Martinez-Navarro et al. [26] reported improved alertness and reaction time but no difference in runners’ cognitive functioning before and after a marathon [26], but there is still a small number of studies focused on the cognitive function of marathon runners.

### 1.3. Brain Structures Engaged during Performing the Tests

Executive functions characterized by intentionality [27] depend on a neuronal circuit in which the prefrontal cortex plays an important role [28]. The dorsolateral part of the prefrontal cortex is active during performance of the Stroop color and word test [29]. The caudate nucleus plays an important role in executive control associated with cognitive elasticity [30]. Short-term verbal memory associated with memorizing digits engages the part of the brain on the border of the middle and superior temporal gyrus and the underlying white matter [31]. Short-term memory may also be associated with the left posterior superior temporal gyrus and sulcus [30], as well as white matter volume in the fronto-parietal regions [32].

### 1.4. Present Study

Findings presented in the literature concerning the mood and cognitive functioning of athletes doing extreme endurance sports are inconsistent. Moreover, there are no data about nighttime cross-country races, and there is still a lack of research focusing on the consequences of endurance training and the psychological aspects of physical activity for marathon runners [33]. This encouraged us to look more closely at this phenomenon. The particularly interesting aspect of this research problem is how taking part in such an extremely physically and psychologically demanding run may impact the cognitive functioning of athletes immediately after finishing it. It can be hypothesized that athletes’ stress levels will be higher before than after the run. Moreover, we assume that the stress experienced by runners before the run will be associated with their performance on cognitive functioning tests. It is difficult to predict the direction of this correlation, as the stress experienced before the race could be related to both the increase and the impairment of attention focus and short-term memory. Assessing the direction of this correlation is the main goal of this study. Given the number of participants, the obtained results will be exploratory and can shed light on the future investigation of differences between nighttime and daytime running.

## 2. Materials and Methods

### 2.1. Participants

The study included 20 experienced runners aged 26–57 (M = 37.29; SD = 7.94) who had M = 7.08, SD = 5.41 (range 3–44) years of experience running. There were 18 men and 2 women. The subjects trained an average of M = 4.96; SD = 1.76 times a week and ran an average of M = 204.25 km, SD = 118.26 km a month. Information about the study was distributed through internet forums for runners and by the event organizers. Purposive sampling was used—participants volunteered to take part in the study. The inclusion criteria for the study concerned active participation in ultramarathon competitions, a minimum of three years of experience in ultramarathon running and written consent to participate in the study. Two of the subjects did not finish the run—one man withdrew from the race after 30 km due to gastrointestinal problems and one woman finished the race after having run a regular marathon (42 km).

### 2.2. Procedure

The protocol of this study was approved by the Independent Bioethics Committee of the Medical University of Gdańsk: NKBBN/434/2018. The Kaszubska Poniewierka run took place on 15 September 2018, starting at midnight and ending at 16.00 (after which time results were no longer recorded). The run itself is considered a qualification run by the International Trail Running Association and the participants gain qualification points to take part in the Ultra-Trail du Mont-Blanc and the Courmayeur–Champex–Chamonix run. The run has a set route, consisting of 95% trail, 80% of which is through forest. Several difficulties await the participants during the race, such as (during the initial phases of the run) running at night on demanding terrain, with sections with fallen trees, steep inclines and declines, roots sticking out of the ground, and narrow and steep paths (single tracks) on a cliff just over water. The difficulty was enhanced by the fact that the most technically demanding part of the run took place at night. There were 175 participants in the race (of whom 20 took part in this study). The race was 100 km long and 113 runners finished within the time limit. The winner finished the race in 09:45:03, after which he went on to run another cross-country, of 51 km, which he also won. The last participant finished the run in 15:57:40. The runners who took part in this study proceeded to the research stand near the start line after collecting their race pack. They first completed the UWIST Mood Adjective Checklist and then, with another researcher, they completed the Stroop test and the memory test (pre-test). Then they proceeded to the start line. The time difference between the first participant and the last participant completing the tests was about 35 min, with the last completing the tests 10 min before the start. Then after about 10 h and having moved the research stand to the finish line, a researcher invited the finishers to the post-test. The tests were completed in the same order and, after the psychological measurements, blood was taken and anthropometric as well as strength measurements were performed (for the purposes of another study).

### 2.3. Methods

The UWIST Mood Adjective Checklist was used to measure the mood of participants [34], in its Polish adaptation [35]. During the test, the runners assessed the degree to which their present mood was described by each of the 29 listed adjectives (on a scale from 1 to 4). The final score is represented by three dimensions: energetic arousal (EA), tense arousal (TA) and hedonic tone (HT). High levels of energetic arousal correspond with such feelings as being restful, energetic and vigorous, and high scores of tense arousal correspond to being stressed, anxious or tense. Hedonic tone is associated with being cheerful, satisfied, and happy (high scores), or concerned, depressed and sad (low scores; [35]). Before completing the test, the athletes were asked about any potential situations unrelated to training that may modify their mood, but no such factors were declared.

Executive functions and short-term memory were measured using the following tools.

The Stroop color and word test assesses executive functioning associated with goal-oriented activity [27], including cognitive elasticity and the ability to inhibit cognitive interference, which is strongly related to control over one’s own behavior, e.g., before making an impulsive response [28], as well as to working memory and the ability to focus one’s attention.

The “Forward digit span” subtest from the Wechsler Adult Intelligence Scale assesses short-term memory of verbal material by getting the subject to repeat a series of digits [36]. The temporary retention of the presented material is a characteristic of short-term memory [37].

### 2.4. Statistical Analysis

Statistical analyses were conducted using Statistica 13.0 software (TIBCO Software Inc.). In order to evaluate the differences in the studied variables, non-parametric tests of differences for independent groups were used (sign test). The non-parametric Spearman’s rank-order test was used in order to measure the correlation.

## 3. Results

**Figure 1:**
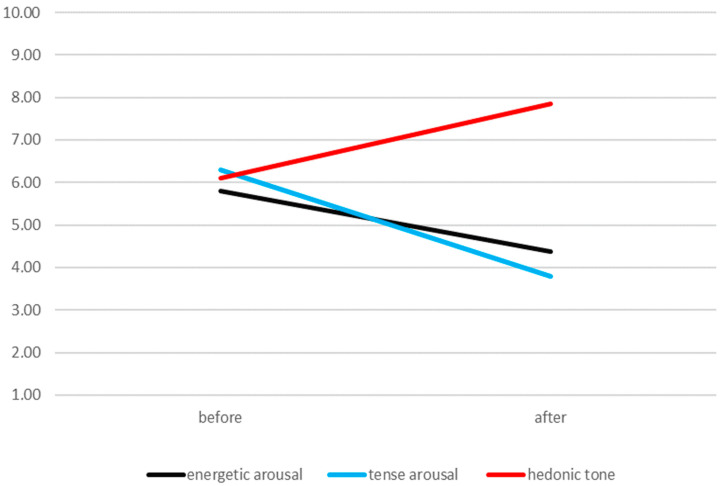
Changes in mood before and after running 100 km.

The first step in the study was to assess the differences between mood before and after the ultramarathon. Before the run, participants exhibited average levels of all of the measured dimensions of mood. On the other hand, after the run, energetic arousal and tense arousal oscillated around low scores, while in the case of hedonic tone, the average scores were high. Participants’ sten scores for mood dimensions are presented in the *y*-axis in Figure 1.

Percentage distributions of mood before and after the run are presented in detail in Table 1.

The only significant difference was in tense arousal (TA). TA before the run (M = 19.85; SD = 4.82) turned out to be significantly higher than TA after the run (M = 12.80; SD = 4.44); Z = 2.46, *p* = 0.014.

The next part of the analyses regarded reaction times on the Stroop color and word test. The reaction times of participants before and after the run are presented (in seconds) in Table 2.

All of the mean reaction times were shorter after the run, but this difference was only significant for Trial 1 (reading the names of colors written in black font).

Then we compared the number of numbers remembered by the participants before and immediately after the run. Before the run, participants remembered on average M = 7.32 (SD = 1.63) numbers and after the run they remembered M = 8.18 (SD = 1.47). This difference was statistically significant; Z = 2.77, *p* = 0.006.

Next, the potential relationship between the mood of participants and their cognitive functioning was analyzed. Spearman’s rank-order correlation analysis revealed one significant relationship. The higher the tense arousal experienced by the participants before the run, the faster they solved the first trial in the color and word test; r = –0.47, *p* = 0.044. No other significant relationships were observed.

Similarly, no relationship was observed between experience in running ultramarathons, number of training sessions, or the age of study participants and their mood and cognitive functioning.

The analysis of correlations between mood and cognitive functioning also did not show any correlations with the participants’ results. Interestingly, time achieved in the ultramarathon did not correlate with the experience or age of the participants, but only with the number of kilometers run per month; r = −0.54, *p* = 0.046.

## 4. Discussion

The presented study aimed to show changes in ultramarathoners’ mood and cognitive functioning before and after a 100 km cross-country run. The race in question took place largely at night, on difficult terrain, and so it was hypothesized that tense arousal would be high before the start and that it would drop after finishing the race. The results largely agree with this assumption: participants’ levels of tense arousal were indeed significantly different before and after the run. This is in line with previous research [11,12,15]. It was assumed that before the run most participants would be characterized by high levels of tense arousal, and this was indeed observed. After the race, participants who exhibited high levels of tense arousal were in a definite minority. This result can definitely be explained by the stress associated with fears regarding the course of the run, possibility of injury, or the anticipated pain [9]. The fact that no correlation was found between tense arousal and experience with running ultramarathons could be explained by the fact that the character of stress may differ between the more and less experienced runners. Athletes who have previously completed fewer ultramarathons may be more afraid of the unknown and may be unsure as to how their body will react to such a strain. In turn, experienced runners remember the pain and exhaustion of their previous runs, and often their fears are associated with unwillingness to experience these states again. Moreover, it is notable that the levels of tense arousal in the runners before the nighttime race were two stens higher than in runners who took part in our previous study, in which participants ran 100 km on a track [10]. It is not surprising that night ultramarathons are more of a mental challenge than a 100 km run on a flat, well-prepared track. Similarly, it is not surprising that hedonic tone increased after finishing the run. Despite the fact that a quantitative analysis did not find this difference to be statistically significant, qualitatively this increase was significant. Probably, irrespective of the experience, finishing an ultramarathon generates satisfaction and a sense of self-efficacy. The relatively high results for energetic arousal in runners after finishing the ultramarathon may be surprising. However, it should be noted here that this could be explained by the runner’s high effect [38,39]. The endogenous secretion of opioids during prolonged effort is not only associated with mood improvement but also with reductions in the perception of pain, which just after finishing the race may lead to an impression that the runner feels like they are in a good condition. If the tests were done at a longer time interval after finishing the run, much lower results on this scale might be expected.

Analysis of the cognitive functioning of runners, especially after the run, yielded some interesting results. The incredible effort that the runners have expended might suggest that it would be difficult for them to even undertake cognitive tasks, let alone to actually perform well at these tests. Nevertheless, we found that results on the color and words test after the run were very similar to those before the run, and in the case of the first trial even higher. The difference on the third trial was on the border of statistical significance. As shown by Wollseiffen et al. [40], prolonged physical fatigue may result in a reduction of psychomotor and neuromuscular function, however it does not necessarily have to be associated with mental fatigue and a reduction in cognitive functioning. Nevertheless, their study showed a decrease in the activity of beta waves in the frontal cortex during a six-hour run, which in theory should lead to a decrease in performance on the Stroop test. The observed result may be explained by the effect of learning from the repeated taking of the measures, though the improvement of performance that also occurred on the memory test could suggest that a different underlying process is responsible for the observed results. The other way of explaining those results may come from the fact that an efficient respiratory system contributes to a good physical condition, has an impact on the proper functioning of the brain, and may contribute to the improvement of cognitive functions. According to the classification of sports, running is described as a sport with a high dynamic component and with a low static component [41,42]. This is also supported by the fact that the mood of marathon runners was better, which could improve executive functions as well.

Improvement in cognitive functioning may also be associated with the nervous system becoming more active, blood acidosis, accumulation of metabolic wastes, and humoral immune response. Moreover, better results after finishing the ultramarathon could be explained by the better efficiency of peripheral motor processes [43]. It is also possible that dopaminergic neurons in frontal lobes increase in size as a result of doing sports, and thus impact executive functions, including inhibition. As prefrontal areas are also associated with working memory, it could be conjectured that dopamine also stimulates the activity of cells, which fosters greater short-term memory capacity. However, this potential explanation has to be considered with caution, as empirical evidence in this area is scarce [43].

### Limitations

The number of participants is the main limitation of this study, making conducting more advanced statistical analyses impossible. This is because ultramarathoners are a very specific, elite group. Undertaking such a run is enough of a strain, and most runners would not agree to take part in research engaging cognitive processes. This limitation makes it impossible to show the relationship between cognitive functioning and the level of fatigue resulting from running in the cause-and-effect approach (e.g., SEM analysis). The other limitation regards the lack of a clear comparison between daytime and nighttime runs—it would definitely show the differences between those two types of events. Thus the obtained results may be treated as an exploratory finding. Moreover, it is worth noting that the pre-test was done at night, with artificial light, which could influence the results—especially those of the Stroop test. However, it should be noted that none of the participants complained about difficulties in reading the test instructions. Electroencephalography (EEG) measurements could undoubtedly enrich the empirical material of the study, but it would be a significant additional burden for the runners to take part in such measurements.

## 5. Conclusions

The main conclusion of the presented study is that taking part in an extremely strenuous nighttime ultramarathon is associated with the hypothesized mood changes. Our results suggest that tense arousal may be higher before nighttime ultramarathons than before races which take place during daytime. Moreover, the Stroop test and the digit span test showed a post-race improvement in some of the trials, which may be associated with several factors. Deeper research is needed to explain the mechanism underlying the better cognitive functioning associated with extreme endurance sports.

## Figures and Tables

**Table 1:** Mood before and after the run.

	Intensity	Before		After		*p*
		%	n	%	n	
Energetic arousal	Low	30.00%	6	52.63%	10	0.151
	Average	25.00%	5	21.05%	4	0.767
	High	45.00%	9	26.32%	5	0.216
Tense arousal	Low	15.00%	3	68.42%	12	0.002
	Average	35.00%	7	26.32%	5	0.542
	High	50.00%	10	5.26%	1	0.002
Hedonic tone	Low	20.00%	2	0.00%	0	0.039
	Average	35.00%	7	10.53%	2	0.063
	High	45.00%	9	89.47%	16	0.036

**Table 2:** Stroop color and word test results before and after the run.

	Before the Run (n = 20)		After the Run (n = 18)		Difference	
	M	SD	M	SD	*Z*	*p*
Trial 1	4.13 (s)	0.84 (s)	3.74 (s)	0.58 (s)	2.91	0.004
Trial 2	3.83 (s)	0.60 (s)	3.66 (s)	0.48 (s)	0.97	0.332
Trial 3	10.47 (s)	1.77 (s)	9.46 (s)	2.90 (s)	1.94	0.052
